# Bimekizumab plus topical photochemotherapy effective in palmoplantar pustulosis in a patient with hidradenitis suppurativa

**DOI:** 10.1111/ddg.15986

**Published:** 2025-10-18

**Authors:** Neda Cramer, Johannes Mohr, Michael P. Schön, Rotraut Mössner

**Affiliations:** ^1^ Department of Dermatology Venereology and Allergology University Medical Center Göttingen, Germany

**Keywords:** Bimekizumab, Palmoplantar pustulosis, Psoriasis

Dear editors,

Palmoplantar pustulosis (PPP) is a chronic, relapsing inflammatory skin disease characterized by sterile pustules, erythema, and hyperkeratosis on the palms and/or soles. It causes pain and itching, and can impair quality of life.^1^, ^2^ The treatment of PPP is challenging, and standardized treatment guidelines are lacking. Bimekizumab, a dual inhibitor of interleukin (IL)‐17A and IL‐17F, is approved for treating plaque psoriasis, psoriatic arthritis, axial spondyloarthritis and hidradenitis suppurativa (HS).^3^ It has also emerged as a promising therapeutic option for PPP.^4^ Here, we present a case study of a patient with severe PPP and HS who was successfully treated with bimekizumab combined with topical phototherapy with psoralen plus ultraviolet A (PUVA).

A 62‐year‐old Caucasian woman presented with HS and PPP. The HS had manifested ten years earlier and had been treated surgically twice, and showed an insufficient response to six months systemic therapy with clindamycin and rifampicin (600 mg each per day). The patient presented with Hurley stage 2 HS in the inguinal and genital region. Itchy, sharply defined erythematosquamous plaques and pustules were present on her palms and soles that had appeared six months earlier (Figure 1a), prior to the initiation of any systemic therapy for HS. Neither plaque psoriasis nor joint pain were present. There was no family history of skin diseases. She was a current smoker with a 15‐pack‐year history. Her BMI was 30.5, and her other comorbidities were arterial hypertension, a C7/8 disc prolapse, and hypothyroidism, which were treated with candesartan and L‐thyroxine. Daily topical therapy with mometasone or clobetasol did not result in sufficient improvement, and flare‐ups continued to occur. To treat the HS, we initiated bimekizumab therapy at a dose of 320 mg every two weeks for a period of 16 weeks, followed by a maintenance dose every four weeks. Additionally, the approach aimed to effectively manage PPP, supported by promising, though limited, existing data.^4^ At this point, the disease duration of PPP was 21 months. However, due to a misunderstanding, bimekizumab was administered every four weeks instead of every two weeks as approved for HS.

**FIGURE 1:**
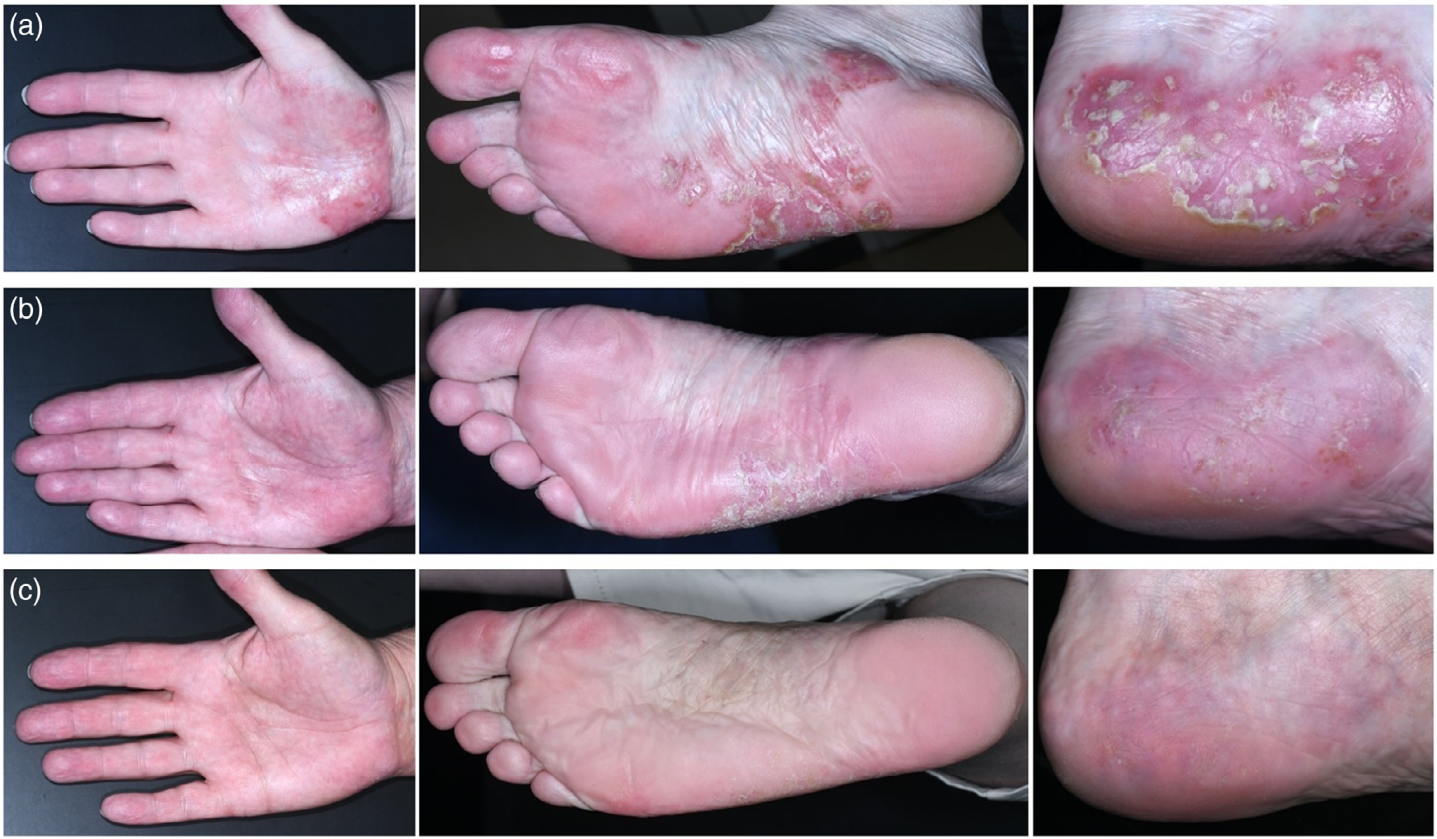
Clinical images of the right palm, right sole and left heel (a) before, and (c‐b) after the start of bimekizumab combined with phototherapy with psoralen plus ultraviolet A (b: after one 320 mg dose of bimekizumab 4 weeks after the start of therapy, c: after a total of three 320 mg doses of bimekizumab 13 weeks after the start of therapy).

Before therapy, the itching of the palms and soles was rated 10 out of 10 on the Numerical Rating Scale (NRS). The Palmoplantar Pustulosis Area and Severity Index (PPPASI) was 11.2 (Figure 1a), and the Dermatology Life Quality Index (DLQI) was 19, indicating that the skin diseases significantly impacted her quality of life. During bimekizumab therapy, topical PUVA was carried out three to four times per week for eight weeks, followed by twice per week. 8‐methoxypsoralen was applied to the palms and soles 30 minutes before irradiation, and topical PUVA was started with 0.50 joules(J)/cm^2^ and was continued by the patient's local dermatologist. Mometasone cream was gradually reduced over the first three weeks and then discontinued. After four weeks of bimekizumab therapy, PPP improved markedly, with the PPPASI falling to 5.5 (Figure 1b). After 13 weeks, the PPPASI decreased further to 0.9 (Figure 1c), and the DLQI was 0. The only adverse event was a febrile gastrointestinal infection that occurred after 11 weeks of therapy and resolved within a week. The inguinal HS lesions remained severe and inflamed during treatment. Even before initiation of therapy, erythematous papules and pustules were present in the area of the HS lesions, which were clinically most consistent with folliculitis. Mycological diagnostics were negative. Topical treatment with a combination preparation of halometasone and triclosan led to an improvement.

The therapeutic challenges of PPP derive from its recalcitrant nature and limited response to conventional treatments. IL‐17A inhibitors have shown some potential for treating PPP. While a phase 3 clinical trial with the IL17A blocker secukinumab failed to meet its primary end point, brodalumab, a IL‐17 receptor A inhibitor, demonstrated significant efficacy in a phase 3 clinical trial.^5^, ^6^, ^7^ Bimekizumab has a much higher binding affinity to IL‐17A than secukinumab.^8^ Studies have also shown that both IL‐17A and IL‐17F are elevated in PPP lesions, which could contribute to the recruitment of neutrophils and amplified local immune responses.^9^, ^10^ Therefore, bimekizumab may suppress the inflammatory cascade in PPP more effectively than secukinumab. The combination of topical PUVA and bimekizumab in this case targets distinct pathogenic pathways and may offer enhanced efficacy through synergistic effects. Prior studies have shown improved outcomes when PUVA is combined with systemic agents like acitretin or methotrexate.^11^, ^12^ While data on PUVA with IL‐17 inhibitors in PPP are lacking, existing evidence supports the plausibility of such combinations.

The effect of bimekizumab on HS in our patient cannot be adequately assessed at this stage, as every second dose was not administered during the dose escalation period up to week 13. This resulted in fewer doses being administered overall (only three 320 mg doses instead of six). In phase 3 clinical trials, the primary efficacy outcome was measured at week 16, with many patients showing meaningful improvement as early as weeks 4–12.^13^ The data also indicate that therapeutic response continues to increase beyond this endpoint.^13^ Based on this evidence, we plan to continue therapy and perform the re‐evaluation of efficacy no sooner than week 24, using the licensed dose. In case of insufficient treatment response to bimekizumab, adalimumab may represent an alternative, in line with its licensed indication for HS, offering the potential to manage both HS and PPP.^14^, ^15^, ^16^

Regarding PPP, our case report demonstrates the excellent efficacy of bimekizumab combined with PUVA therapy, with a rapid onset of action. After four weeks, there was a 50% improvement of the skin lesions and PPPASI, and almost complete clearance was achieved after 13 weeks. To date, there are no published data on the treatment of PPP with bimekizumab except for one case series.^4^ In this recently published, retrospective multicenter case series of 21 patients, bimekizumab demonstrated substantial efficacy in patients with PPP or palmoplantar plaque psoriasis with pustules. Notably, 17 out of 21 patients achieved complete remission within one to four months.^4^

Together with our case report, these findings suggest that bimekizumab in combination with topical PUVA may offer a valuable new approach for managing severe and treatment‐resistant PPP.

## CONFLICT OF INTEREST STATEMENT

J.M. has been an advisor and/or received grants and/or participated in clinical trials of the following companies: Abbvie, Allmirall, Biogen IDEC, Böhringer‐Ingelheim, Celgene, Janssen‐Cilag, Leo Pharma GmbH, Lilly, MSD SHARP & DOHME, Novartis Pharma, Pfizer and UCB.

R.M. has been an advisor and/or received speakers’ honoraria and/or received grants and/or participated in clinical trials of the following companies: AbbVie, Amgen, Almirall, Biogen IDEC, Böhringer‐Ingelheim, Celgene, Janssen‐Cilag, Leo Pharma, Lilly, Moonlake, MSD SHARP & DOHME, Novartis Pharma, Pfizer and UCB.

M.P.S. has been an advisor and/or received speakers’ honoraria and/or received grants and/or participated in clinical trials of the following companies: AbbVie, Almirall, Biogen, Boehringer‐Ingelheim, BMS, Celltrion, Janssen‐Cilag, Leo, Lilly, Novartis, Scinai, UCB.

N.C. reports no conflicts of interest.
